# Educational applications of metaverse: possibilities and limitations

**DOI:** 10.3352/jeehp.2021.18.32

**Published:** 2021-12-13

**Authors:** Bokyung Kye, Nara Han, Eunji Kim, Yeonjeong Park, Soyoung Jo

**Affiliations:** 1 Global Policy and Research Section, Digital Education Policy Division, Korea Education and Research Information Service, Daegu, Korea; 2 Department of Early Childhood Education, College of Humanities and Social Sciences, Honam University, Gwangju, Korea; Hallym University, Korea

**Keywords:** Augmented reality, Communication, Educational personnel, Medical education, Virtual reality

## Abstract

This review aims to define the 4 types of the metaverse and to explain the potential and limitations of its educational applications. The metaverse roadmap categorizes the metaverse into 4 types: augmented reality, lifelogging, mirror world, and virtual reality. An example of the application of augmented reality in medical education would be an augmented reality T-shirt that allows students to examine the inside of the human body as an anatomy lab. Furthermore, a research team in a hospital in Seoul developed a spinal surgery platform that applied augmented reality technology. The potential of the metaverse as a new educational environment is suggested to be as follows: a space for new social communication; a higher degree of freedom to create and share; and the provision of new experiences and high immersion through virtualization. Some of its limitations may be weaker social connections and the possibility of privacy impingement; the commission of various crimes due to the virtual space and anonymity of the metaverse; and maladaptation to the real world for students whose identity has not been established. The metaverse is predicted to change our daily life and economy beyond the realm of games and entertainment. The metaverse has infinite potential as a new social communication space. The following future tasks are suggested for the educational use of the metaverse: first, teachers should carefully analyze how students understand the metaverse; second, teachers should design classes for students to solve problems or perform projects cooperatively and creatively; third, educational metaverse platforms should be developed that prevent misuse of student data.

## Introduction

### Background

The metaverse concept first appeared in 1992 in the science fiction novel Snow crash by American novelist Neal Stephenson. The characters in *Snow crash* become avatars and work in a 3-dimensional (3D) virtual reality, and this 3D virtual reality is called the metaverse. The metaverse refers to a virtual reality existing beyond reality. It is a compound word of “meta”, meaning transcendence and virtuality, and “universe”, meaning world and universe. This term refers to the digitized earth as a new world expressed through digital media such as smartphones and the internet [1]. After the concept of the metaverse appeared, extensive efforts and research were carried out to make the metaverse a reality. The Acceleration Studies Foundation (ASF), a representative metaverse research organization, announced the metaverse roadmap in 2006. It presented the concept of the metaverse and the 4 types of the metaverse and proposed thinking about the metaverse as a connection point or combination of the real world and virtual reality.

Go et al. [2] defined the metaverse as “a 3D-based virtual reality in which daily activities and economic life are conducted through avatars representing the real themselves.” Here, daily activities and economic life are extensions of reality, and it is seen that the real world is combined with the virtual space, and reality is expanded into the virtual space. In other words, the avatar in the metaverse is identified with one’s real self. The avatar engages in social, economic, and cultural activities in the metaverse world. In addition, Lee [3] stated that “metaverse means a world in which virtual and reality interact and co-evolve, and social, economic, and cultural activities are carried out in it to create value.” This is not a simple combination of the world and virtual reality, but an interaction; furthermore, the metaverse can denote a world in which daily life and economic activities are conducted in a unified way.

As the metaverse began to be introduced into present life rapidly, some metaverse applications have already been used in education. Therefore, it is necessary to understand the concept and types of the metaverse and examples of its educational applications.

### Objectives

This review aims to define the 4 types of the metaverse and explain the potential and limitation of the educational applications of the metaverse. Specifically, the characteristics of the 4 types of metaverse are described with examples. The merits of applications of the metaverse in the educational field are presented. Furthermore, the limitation and disadvantages of the use of the metaverse are discussed. Thus, this review will be able to provide basic insights into the concept of the metaverse for applying it in education.

## Four types of the metaverse

**Fig. 1.**
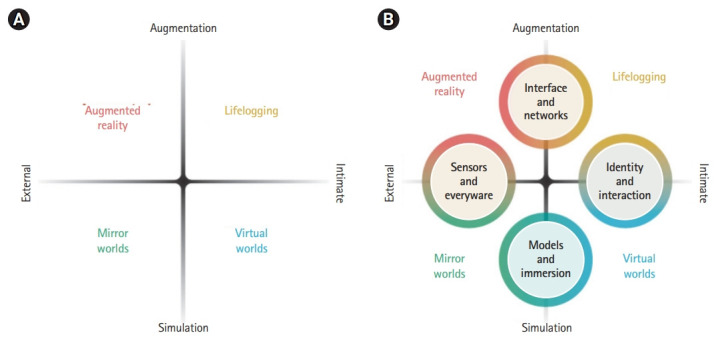
(A, B) Diagram of 4 types of the metaverse [4].

In the ASF’s metaverse roadmap, 2 axes were presented to explain the types of the metaverse [4]. One is ‘augmentation versus simulation’, and the other is ‘intimate versus external’ (Fig. 1). Augmentation technology refers to a technology that adds a new function to an existing real system. In the metaverse, augmentation technology superimposes further information on the physical environment that we perceive. Simulation technology, which contrasts with augmented technology, refers to technology that provides a unique environment by modeling reality. Simulation in the metaverse includes various techniques for realizing the simulated world as a place for interaction. In short, augmented technology and simulation can be divided according to whether the information will be implemented in physical reality or virtual reality.

Meanwhile, the metaverse is divided into an inner world and an external world. The inner world focuses on the identity and behavior of an individual or object. Technology is used to achieve completion of the inner world in the metaverse. Individuals or things act using an avatar or digital profiles or act directly in the system, whereby the user has agency in that environment. In contrast, the external world usually focuses on aspects of external reality centered on the user, the subject of the metaverse. Therefore, it includes technology related to displaying information about the user’s surrounding world and how to control it. These internal and external frameworks become another axis for dividing applications based on whether metaverse technology is focused on the user’s inner world or the surrounding world.

The metaverse roadmap categorizes the metaverse into 4 types: augmented reality, lifelogging, mirror world, and virtual reality based on these 2 axes. Table 1 summarizes the definitions, characteristics, application fields, and use cases of each type.

### Augmented reality

**Fig. 2.**
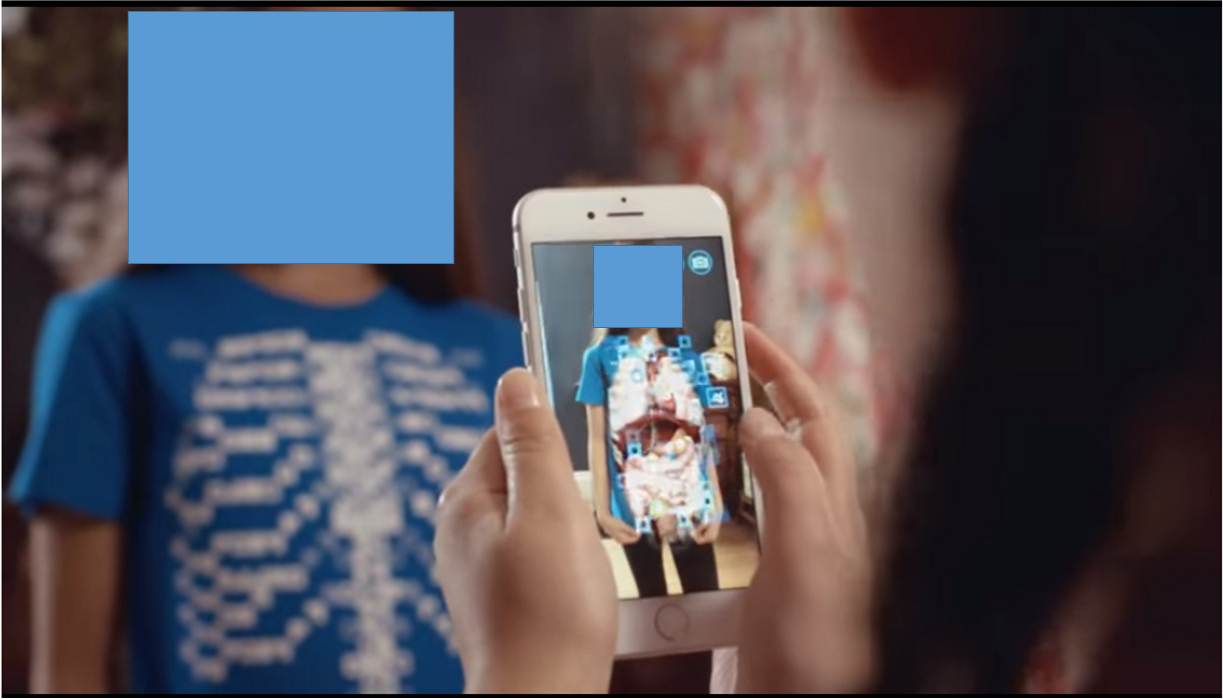
Virtuali-Tee: augmented reality T-Shirt [7].

**Fig. 3.**
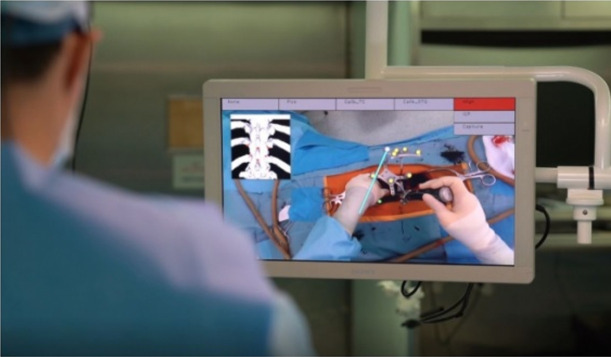
Augmented reality-based spine surgery platform [8].

Augmented reality is a type of augmentation of the external world. It refers to a form of technology that expands the real physical world outside an individual by using a location-aware system and interface with added and layered networked information on spaces we encounter daily [4]. The interfaces that augment the world are divided into Global Positioning System (GPS)-based, marker-based, and see-through-based [5,6]. By utilizing the built-in GPS and Wi-Fi in a mobile device, augmented reality provides linkage information suitable for the user’s location information or recognizes a marker in a QR (quick response) code to augment already extant information. In addition, the real world and virtual graphics can be mixed and viewed in real-time through glasses or lenses. Augmented reality has been evaluated to be effective in learning material that is difficult to observe directly or explain in text, fields that require continuous practice and experience, and fields with high costs and high risk [5]. For example, Cruscope’s Virtuali-Tee is an augmented reality T-shirt that allows students to examine the inside of the human body as if it were an anatomy lab [7] (Fig. 2). Augmented reality simulation content as a representative educational case is related to augmented reality. Simulation plays a role in linking abstract visuals to concrete objects by connecting the context of the real world and virtual objects. In the medical field, various examples of augmented reality technology are emerging. Recently, a research team in a hospital in Seoul developed a spinal surgery platform that applied augmented reality technology in collaboration with university laboratories. This platform uses a real-time projection of a pedicle screw used for spinal fixation on a human body structure as an overlay graphic based on augmented reality [8]. In addition, based on this technology, a spinal surgery education program will be developed to implement an effective education system that can be applied to actual surgery (Fig. 3).

### Lifelogging

**Fig. 4.**
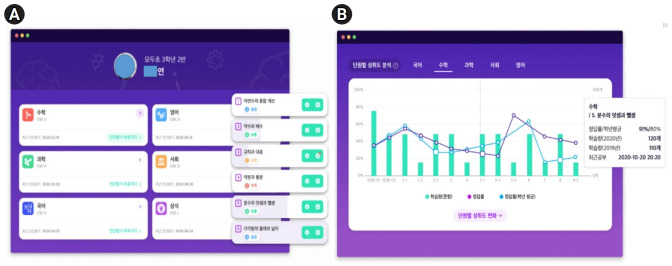
(A, B) Artificial intelligence analysis screen for all subjects and distribution table of achievement changes by unit [9].

Lifelogging is a type of augmentation of the inner world. In the world of lifelogging, people use smart devices to record their daily lives on the internet or smartphones. Typical examples of lifelogging include Twitter, Facebook, and Instagram. Recently, there have been services that utilize biometric information stored through wearable devices in the medical field. Some devices link sensors such as Nike Plus to record the amount of exercise or location. This is also a kind of lifelogging. As a representative example, the Classting artificial intelligence (AI) system in Korea is an online class community application called an educational social networking service (SNS). In particular, Classting AI analyzes students’ learning achievements and provides customized learning by level in all subjects [9] (Fig. 4).

### Mirror world

The mirror world is a type of simulation of the external world that refers to an informationally enhanced virtual model or “reflection” of the real world [4]. The mirror world is a metaverse where the appearance, information, and structure of the real world are transferred to virtual reality as if reflected in a mirror. However, the expression “efficient expansion” is more appropriate than describing these systems as reproducing the real world [1]. All activities in the real world can be done through the internet or mobile applications, and a mirror world metaverse is a place that makes life in the real world convenient and efficient. Examples of representative mirror worlds used in education include “digital laboratories” and “virtual educational spaces” created in various mirror worlds.

#### Digital labs

**Fig. 5.**
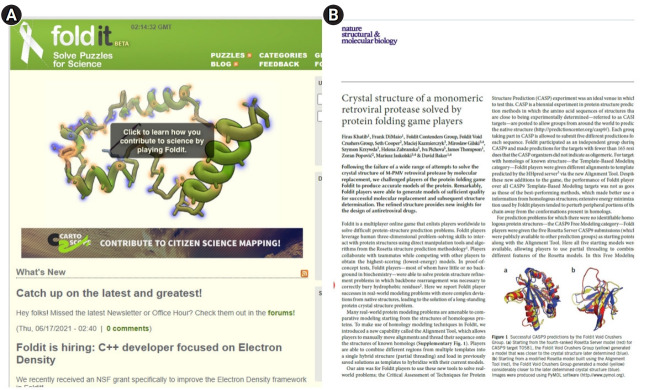
(A, B) Foldit platform as a mirror world example [10].

The mirror world metaverse was further activated by the coronavirus disease 2019 (COVID-19) pandemic. In other words, the biggest contributor to enabling the mirror world is the user. Users meet and play games with physically distant people in the mirror world and perform meaningful tasks. The Foldit platform provides an opportunity for all participants to contribute to scientific research through games. David Baker’s team at the University of Washington, which studies protein structure, has used this digital lab to have people fold protein amino acid chains. Through this game, where the protrusion structure matches well, and the player gets points and ranks up if they succeed, the protein structure is found for an AIDS (acquired immunodeficiency syndrome) treatment, and the achievement of 60,000 participants in 10 days was described in a journal publication [10] (Fig. 5).

#### Virtual educational spaces

**Fig. 6.**
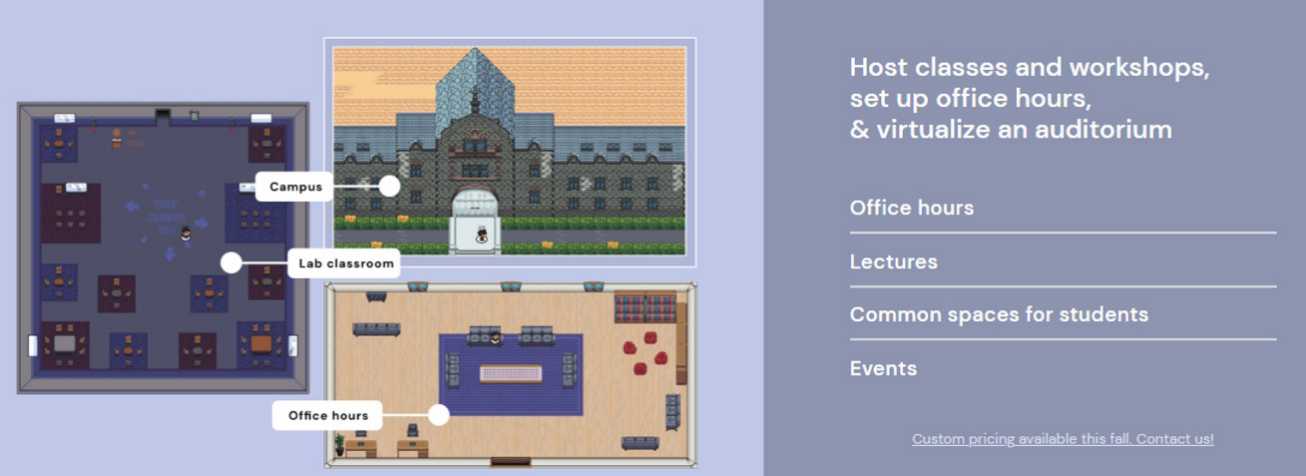
Classroom in Gathertown [11].

A representative example of the mirror world is video conferencing systems such as Zoom, Webex, Google Meet, and Teams. These video conferencing systems are playing the role of the classroom in the real-time operation of non-face-to-face remote classes in the post-COVID-19 era. Gathertown is an online video conferencing platform that supports conversation and business in a virtual space [11]. Its main functions include chatting, interworking with external links, and decorating spaces (Fig. 6). The mirror world metaverse has great educational potential as a way to efficiently expand the information and functions required for learning while showing the real world exactly as if reflected in a mirror [1].

### Virtual reality

Virtual reality is a type of the metaverse that simulates the inner world. Virtual reality technology includes sophisticated 3D graphics, avatars, and instant communication tools. It is a world where users feel that they are entirely in a virtual reality. Virtual reality is often described as the other end of the spectrum containing mixed reality and augmented reality [12]. However, virtual reality makes us see a flat image in 3 dimensions based on the working principle of our eyes [6]. It is also characterized as an internet-based 3D space that multiple users can access simultaneously and participate by creating an avatar that expresses the user’s self [13].

In this virtual reality metaverse, the space, cultural background, characters, and institutions are designed differently from in reality. The avatar that acts on behalf of the user explores a virtual space with AI characters, communicates with other players, and achieves the goal. Virtual reality is also called the metaverse in the narrow sense in that the real body moves, touches something, and daily and economic activities take place in the virtual space. Zepeto and Roblox are examples of virtual reality [14,15]. Zepeto is a 3D avatar-based interactive service that has recently appeared, and Roblox is a platform where anyone can create virtual reality and create games by themselves to enjoy with friends and engage in various creative experiences [16].

**Fig. 7.**
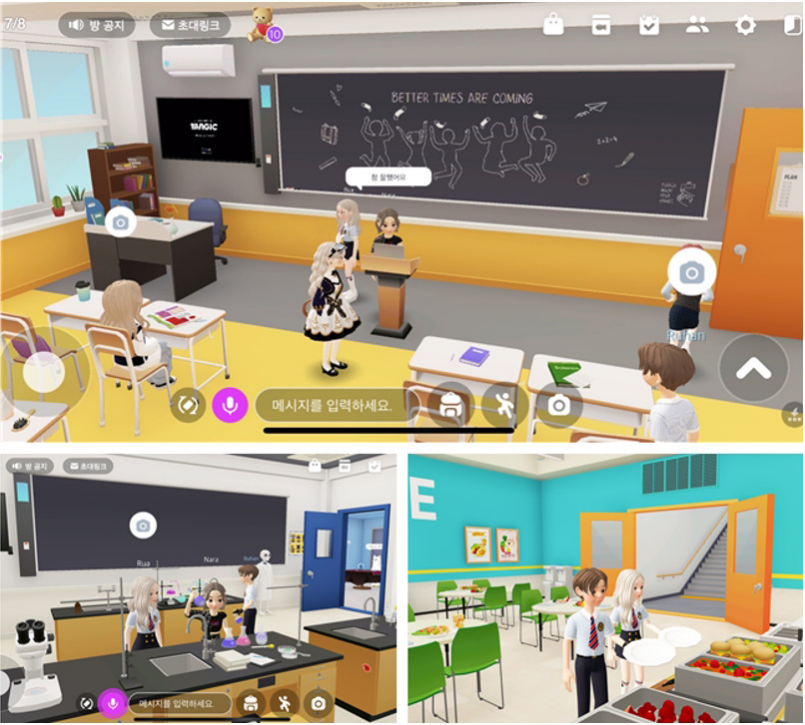
Class 2 map in Zepeto. From Snow Corp. Zepeto [Internet]. Seongnam: Snow Corp.; c2021 [cited 2021 Nov 29]. Available from: https://zepeto.me/ [14].

Zepeto is an augmented reality avatar service operated by Naver Z, and is a representative metaverse platform in Korea. Zepeto, launched in 2018, creates a “3D avatar” using facial recognition, augmented reality, and 3D technology to communicate with other users. It allows users to experience various virtual realities. When anyone takes a picture or loads an image saved in his or her smartphone, a character that resembles the user is created through AI technology. They can customize skin color, features, height, facial expression, gestures, and fashion style as they like. Since the SNS function is also incorporated, it is possible to follow other people and to exchange messages by text or voice [14]. Various activities such as games and educational role-plays can be performed through multiple maps. For example, a teacher can select a classroom map, open a room, invite students, and interact with each other by voice or message on the classroom map [14] (Fig. 7).

**Fig. 8.**
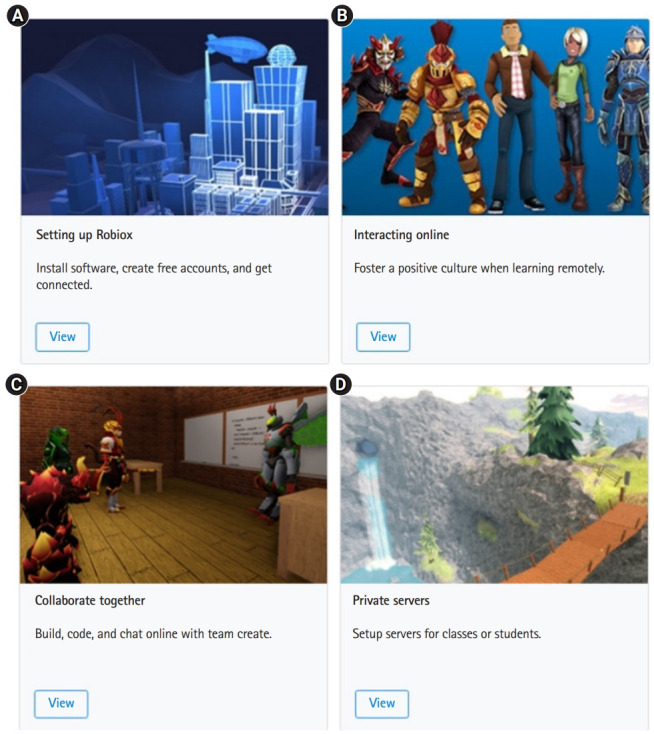
(A–D) Roblox remote education tool (https://education.roblox.com/ko-kr/resources/roblox-remote). From Roblox Corp. Roblox [Internet]. San Mateo (CA): Roblox Corp.; c2021 [cited 2021 Nov 29]. Available from: https://www.roblox.com [15].

Roblox is a virtual reality platform launched in 2006, where one can create one’s own space and enjoy games in real-time. Lee and Han [17] explain that Roblox is a “second real world” in which the virtual currency “Robux” is used, and the economic ecosystem is completed. It is characterized by users being able to make games on their own in virtual reality using the Lego-shaped avatar or to enjoy games made by others [15] (Fig. 8).

## Convergence and complexity of the metaverse

We have looked at the characteristics and examples of each of the 4 types of the metaverse. The metaverse can be organized as a space in which the real world is augmented by virtual reality. The real world is connected to virtual reality, the real world is replicated in virtual reality, or the virtual reality becomes another world. From a functional point of view, the metaverse integrates information retrieval, SNS, and game elements. From an evolutionary point of view, the metaverse is a mixture of the internet with 5G and virtual convergence technology, reflecting a world that has been spread and developed in response to the COVID-19 pandemic. From a technical point of view, the metaverse is a complex of virtual reality technologies [2]. Socially, it is a space where members of the digital native generation leave traces in their daily life and economic life with their various appearances (personas, avatars) in the 3D-based internet world. Table 2 summarizes the characteristics and educational implications of each type of metaverse discussed above.

The core of the metaverse can be seen as “extension” and “connection.” The 4 types of the metaverse were developed independently in the beginning. Nonetheless, they have recently been evolving into a new kind of convergence/composite service by interacting while breaking down boundaries. For example, Ghost Pacer, presented as lifelogging, combines augmented reality and lifelogging service [3]. In addition, the virtual conference service Roomkey [18] introduced in a mirror world is an example of the fusion of lifelogging, the mirror world, and virtual reality. Existing non-face-to-face meetings such as video conferences and webinars have the disadvantage that there is no proper way to measure performance other than the number of simultaneous users. In this service that combines virtual reality and lifelogging concepts in a mirror world, all activities such as meetings and networking are measured after the performance.

## Characteristics of the virtual reality metaverse in education

Among the 4 types of the metaverse, the most diverse and actively used technology in education is virtual reality. In particular, the recent non-face-to-face (untact) era has been characterized by the frequent the utilization of virtual reality, , which can be accessed from anywhere regardless of distance or space. Go et al. [2] presented 5 characteristics of the virtual reality metaverse that distinguish it from existing platform services as the “5 Cs” as follows: first, as a canon, the space-time of the metaverse is created and expanded by designers and participants together; second, a creator (anyone in the metaverse) can create content; third, as a digital currency, production and consumption are possible through the production of various contents; fourth, the continuity of everyday life is guaranteed through the metaverse; and fifth, the metaverse connects the real and the virtual, connects the metaverse worlds, and connects people (avatars).

Book [19] also suggested 6 characteristics of virtual reality as follows: first, as a shared space, virtual reality should be a space where multiple users can participate at the same time; second, with a graphical user interface, virtual reality is visually expressed and implemented in a 2-dimensional (2D) or 3D environment; third, immediacy means that interactions between users in the virtual reality occur in real-time; fourth, interactivity allows users to interact with various contents of the virtual reality, such as transforming or developing contents; fifth, the virtual reality is persistently maintained regardless of the access of individual users; and sixth, with socialization/community, a community that can perform various social actions in the virtual reality can be formed.

## Potential of the metaverse as an educational environment

The recent metaverse craze has started again, according to the transition to an untact society due to the COVID-19 pandemic. The 4 existing metaverse types—augmented reality, lifelogging, mirror world, and virtual reality—are accelerating the utilization of the metaverse as it evolves into a new type of convergence service. It also breaks down the boundaries between these types of the metaverse. As face-to-face communication becomes difficult due to the spread of COVID-19, activities that were thought to be only possible offline are being converted to virtual reality and are rapidly expanding into various fields such as education, medical care, fashion, and tourism.

### A space for new social communication

Due to the prolonged COVID-19 pandemic, it is not easy to have private meetings of many people or eat together in a restaurant. However, in the metaverse, hundreds of thousands or tens of millions of people can gather to hold a festival or watch a concert by their favorite singer. Virtual reality metaverses such as Roblox and Zepeto provided people who could not go out due to COVID-19 with a new social space for meeting and relaxation.

When schools were closed due to COVID-19 and students could not attend school, the “Classroom Map” among various 3D maps in Zepeto was the most popular. Instead of going to the real classroom, the students went to the Zepeto classroom. They met and communicated with their friends. The idol group *Blackpink* held a virtual fan signing event through Zepeto when they could not hold a fan signing event face-to-face due to COVID-19. *Blackpink* also released a choreography music video for “Ice cream” that they reproduced with their avatar [20] (https://gweb.zepeto.io/user/post/97321663). More than 46 million users attended Blackpink’s virtual fan signing event to get autographs and take selfies with their favorite singers. The idol group *BTS* also held a showcase by releasing the choreography version of the music video for “Dynamite” on Fortnite for the first time [21]. Users who attended the event forgot about COVID-19 and enjoyed the event by dancing together or sharing their impressions.

### A higher degree of freedom to create and share

Some may believe the metaverse to be merely online games, but it is an evolved concept. In online games, service users have no choice but to perform limited missions according to the goals set by the platform provider. However, in the metaverse, ultimately, anything the user wants is possible without a pre-determined mission. Based on the perfect degree of freedom, users can experience various things in the real world, such as study, shopping, performances, exhibitions, and tourism. They can also share events that are difficult to experience easily due to physical restrictions in the real world, such as flying in the sky and going to space. Alternatively, users can get away from their busy lives and enjoy the leisurely life of fishing, picking fruits, or traveling to a friend’s island all day without the task of solving the metaverse. Everything is the user’s choice.

By actively utilizing the characteristics of the metaverse, it will be possible to design learning activities that can expand students’ freedom and experience to an infinite extent. Students will conduct self-directed learning that allows them to explore their questions based on their endless autonomy. They can refer to the ideas of countless people across time and space and take the initiative in finding their original answers.

### Providing new experiences and high immersion through virtualization

The metaverse is attracting attention as an alternative to overcome the limitations of existing 2D-based online and remote classes. It can provide a differentiated experience value from the current internet era due to the complex use of various technologies. Furthermore, the metaverse makes it possible to design a new experience that transcends time and space. Metaverse-based education enables the use of infinite space and data and has the advantage of allowing interaction at the level of face-to-face education [3].

## Limitations of the metaverse in educational application

The metaverse has made users “social connection” possible by providing a place where people with hobbies and interests can gather and communicate even under real-world restrictions such as “social distancing” in response to the COVID-19 pandemic. However, these social connections in the metaverse are weaker than interactions in the real world. In metaverse, rather than showing “me as I am,” information that one does not want to share is deleted to create “me I want to show.” In addition, privacy infringement is also a problem that needs to be considered in social activities in the metaverse, where various information that was not generated in real-world interactions is collected and processed in real-time.

The high degree of freedom, which is an advantage of the metaverse, makes metaverse users more dangerous than users of existing online services and games. The administrator cannot predict all the actions of users due to the high degree of freedom. Due to the essential characteristics of the metaverse—virtual space and anonymity—people’s sense of guilt about crimes is reduced. There is a concern that new crimes that are more vicious and sophisticated than the real world may appear. The ‘I’ participating in the virtual world may have a similar appearance and self as an extension of reality, but may participate as a self with a different appearance and worldview. The term sub-character (additional character) can be interpreted as the concept of an avatar. As life in which the virtual world and reality are combined become commonplace, the degree of freedom in people’s identities is expected to increase gradually in a virtual space where one’s identity is not exposed at all. People can be recognized in a limited way compared to reality. They should be careful because they can be more easily exposed to criminal activities in the metaverse with a higher level of anonymity. In a metaverse that values freedom, the countless amounts of data created and shared by users worldwide cannot be censored one by one. Therefore, there is a possibility that it will become a lawless zone. In this case, caution is needed because it can be a significant risk to young adolescents with little social experience and whose identity has not been established. Furthermore, ethics education for cultivating citizenship in the virtual world will be required.

As the distinction between the virtual and the real world becomes blurred, users may experience confusion regarding their “real me” identity. They may not be able to adapt to virtual reality properly appropriately. If one is too immersed in human relationships in the virtual reality or satisfied with human relationships in virtual reality, there is a danger that neglecting one’s relationships in the real world can make them worse, or it can make it difficult to establish relationships (Table 3).

## Conclusion

**Fig. 9.**
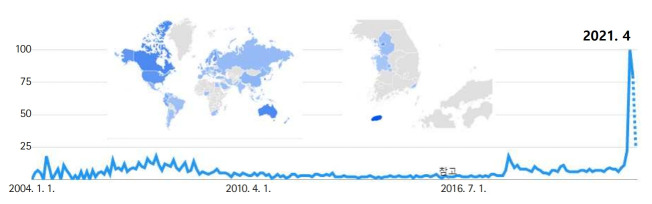
Trends of interest in the metaverse in Google Trends over time. From Google Trends. Metaverse [Internet]. Mountain View (CA): Google; 2021 [cited 2021 Nov 29]. Available from: https://trends.google.com/trends/explore?date=all&q=metaverse [22].

The characteristics of 4 types of the metaverse, the possibility of educational applications, the convergence and complex characteristics of the types of the metaverse, and the potential and limitations of the metaverse for educational applications were described. The metaverse is predicted to change our daily life and economy beyond the realm of games and entertainment. Furthermore, all social, cultural, and economic activities are moving to the metaverse’s new platform. The metaverse has infinite potential as a new social communication space. It provides a high degree of freedom for creation and sharing and provides a unique and immersive experience [22] (Fig. 9). Since the metaverse is expected to grow rapidly during and after the COVID-19 pandemic, it also has risk factors that do not have appropriate regulations. The following are suggested as future tasks for the educational use of the metaverse.

First, it is necessary to carefully analyze how students understand the metaverse, what they want to do there, why they like it, and what value they attach to their avatar in virtual reality. It is necessary to study students’ activity patterns, level of immersion in the metaverse, and its positive and negative effects on students' learning activities.

Second, an effective and attractive aspect of the metaverse is that it allows us to experience events that would be impossible or limited in the real world. However, there is room for uncritically accepting the intentions of content developers or service designers rather than students’ cognitive abilities and imagination. Therefore, instructional designers and instructors who want to utilize the metaverse for education need to properly understand each type of metaverse’s technical characteristics and design classes so that they can solve problems or perform projects collaboratively and creatively.

Third, developing an educational metaverse platform to prevent the misuse of student data is required. Evaluation studies on data collection to support teaching and learning are also required.

## Figures and Tables

**Table 1.** Four types of the metaverse

	Augmented reality	Lifelogging	Mirror world	Virtual reality
Definition	Building a smart environment by utilizing location-based technologies and networks.	Technology to capture, store, and share everyday experiences and information about objects and people.	It reflects the real world as it is, but integrates and provides external environment information.	A virtual world built with digital data
Features	Building a smart environment using location-based technology and networks	Recording information about objects and people using augmented technology	Virtual maps and modeling using GPS technology	Based on interaction activities between avatars that reflect the user’s ego
Applications	Smartphones, vehicle HUDs	Wearable devices, black boxes	Map-based services	Online multiplayer games
Use cases	Pokemon Go, Digital Textbook, Realistic Content	Facebook, Instagram, Apple Watch, Samsung Health, Nike Plus	Google Earth, Google Maps, Naver Maps, Airbnb	Second Life, Minecraft, Roblox, Zepeto

From Lee S. Log in Metaverse: revolution of human×space×time (IS-115) [Internet]. Seongnam: Software Policy & Research Institute; 2021 [cited 2021 Nov 29]. Available from: https://spri.kr/posts/view/23165?code=issue_reports [3]; Smart J, Cascio J, Paffendorf J. Metaverse roadmap: pathway to the 3D web [Internet]. Ann Arbor (MI): Acceleration Studies Foundation; 2007 [cited 2021 Nov 29]. Available from: https://metaverseroadmap.org/MetaverseRoadmapOverview.pdf [4].

**Table 2.** Main technical characteristics of the metaverse and educational implications

Type	Technological characteristics	Educational implications
Augmented reality	- Overlay virtual objects in the real world to make the object 3D and real (e.g., paper birthday cards are augmented to appear as 3D video cards).	- Learn invisible parts visually and 3-dimensionally through virtual digital information, and effectively solve problems
	- Adding fantasy to the thread (e.g., Pokémon Go on the street, Zepeto, which recognizes faces and creates 3D avatar)	- In-depth understanding of content that is difficult to observe or explain in text, and learners can construct knowledge through experience
	- Effectively emphasizing information and promoting convenience (e.g., HUD presented on the car glass)	- Interactive experiences such as reading, writing, and speaking are possible while immersed in the learning context.
Lifelogging	- One’s daily life and thoughts are productively contented and shared through social media and SNS (e.g., blogs, YouTube, Wikis, etc.).	- Review and reflect on one’s daily life, improve the ability to represent and implement information in an appropriate direction, and feedback from others on social networks leads to reinforcement and rewards.
	- Network technology forms relationships with others online, communicates quickly, and records various social activities (Facebook, Band, Twitter, etc.).	- Critically explore various information on the lifelogging platform, and creatively reconstruct information through collective intelligence.
	- Personal activity information is accumulated and analyzed through various sensors of the internet of things and wearable devices to create added value (e.g., health tracking including Nike Plus).	- Reflect on learning and improve it based on analytics data related to learning (e.g., dashboard).
		- Teachers promote learning in a customized direction based on students’ learning log data, provide appropriate support, and prevent dropouts.
Mirror world	- Expanding the real world by combining GPS and networking technology (e.g., Google Earth, various map applications, etc.)	- Overcoming the spatial and physical limitations of teaching and learning, learning takes place in the metaverse of the mirror world.
	- Implementation of the real world into the virtual world as if reflected in a mirror for a specific purpose (e.g., Airbnb, Minerva School, food ordering app, taxi call, bus route guidance, parking lot finder app, etc.)	- Conduct online real-time classes through online video conferencing tools and collaboration tools (Zoom, WebEx, Google Meet, Teams), which are representative mirror worlds.
	- However, it does not contain everything in reality. In other words, it effectively expands the real world to increase fun and play, flexibility in management and operation, and collective intelligence (e.g., Minecraft, Upland, Digital Lab, etc.).	- Through the mirror world, learners can realize “learning by making” (e.g., in Minecraft, students build and restore historical structures—Bulguksa, Gyeongbokgung, Cheomseongdae, Taj Mahal, Eiffel Tower, etc. Users can experience their digital heritage and deepen their understanding of history and culture.
Virtual reality	- Through sophisticated computer graphics work, especially in a virtual environment implemented with 3D technology, users enjoy various games through a seamlessly connected interface (e.g., various 3D games including Roblox).	- Practice can be performed through virtual simulation in environments that are difficult to produce due to high costs and high risk (e.g., fire scenes, flight control, dangerous surgery, etc.).
	- In a space, era, culture, and characters designed differently from reality, they act as avatars rather than their original self, and have multiple personas.	- Users can have immersive experiences of times and spaces that cannot be experienced in reality, such as the past or future era.
	- Chat and communication tools are included in virtual reality to communicate and collaborate with AI characters and others (e.g., multiplayer online games).	- Through 3D virtual world-based games (according to the characteristics and types of designed games), users improve strategic and comprehensive thinking skills, problem-solving skills, and learn skills necessary for the real world.

The technical features are summarized by referring to the content and examples of Kim [1]. 3D, 3-dimensional; HUD, head-up display; SNS, social networking service; GPS, Global Positioning System; AI, artificial intelligence.

**Table 3.** Characteristics of the metaverse, and merits and shortcomings in its educational applications

Metaverse characteristics	Merits	Shortcomings
New social communication space	Even in the case of school closures due to coronavirus disease 2019, students can socially connect beyond the limitations of reality.	When forming a relationship with others, one forms a relationship centered on play that is weaker than the interaction in the real world, and privacy problems occur due to the collection and processing of various personal information.
High degree of freedom	Expanding student autonomy in the learning process by providing experiences from content consumers to creators	Due to the high degree of freedom, platform administrators cannot predict all the actions of users, and they can be exposed to various crimes due to the virtual space and anonymity of the metaverse.
Through virtualization high immersion	By providing a new experience that transcends time and space, it is possible to increase student interest and immersion to expand students’ active participation in learning.	It can cause identity confusion, escape from reality, and maladaptation to the real world for students whose identity has not been established.

## Supplementary materials

Supplementary files are available from Harvard Dataverse: https://doi.org/10.7910/DVN/0KUSGC

Supplement 1. Audio recording of the abstract.
